# Preparation, Mechanical Properties, and Biocompatibility of Graphene Oxide-Reinforced Chitin Monofilament Absorbable Surgical Sutures

**DOI:** 10.3390/md17040210

**Published:** 2019-04-04

**Authors:** Wei Zhang, Bin Yin, Yu Xin, Lei Li, Guanlin Ye, Junxian Wang, Jianfei Shen, Xiao Cui, Qihui Yang

**Affiliations:** 1 College of Fisheries, Guangdong Ocean University, Zhanjiang, Guangdong 524025, China; khzhangwei110@163.com (W.Z.); m15218207856@126.com (B.Y.); xy529_03@163.com (Y.X.); guanlinye2017@163.com (G.Y.); junxianwang95@163.com (J.W.); sjfswbd@163.com (J.S.); cx976605133@163.com (X.C.); 2 Life Science and Technology School, Lingnan Normal University, Zhanjiang 524048, China; lleirun1994@163.com

**Keywords:** graphene oxide, chitin, suture, biocompatibility, mechanical strength

## Abstract

Chitin (CT) is a good material to prepare surgical sutures due to its conspicuous biological characteristics. However, the poor mechanical strength of pure CT sutures limits its application. In order to improve its strength, a composite monofilament absorbable suture was prepared in this study using graphene oxide and chitin (GO-CT) using a green method. FT-IR spectra showed that GO-CT contained the characteristic functional groups of GO and CT, indicating that a GO-CT suture was successfully obtained. With the addition of a small amount of GO (1.6wt% solution) in chitin, the breaking tensile strength, knot strength, and knot-pull strength of the GO-CT suture were significantly improved compared to the CT suture. The biocompatibility of the GO-CT suture in vitro was checked by tetrazolium-based colorimetric assays and no cytotoxicity to L929 cells was found. In vivo, the subcutaneous implantation of GO-CT sutures in the dorsal skin of rats found no abnormalities by hematoxylin-eosin staining. Furthermore, there were no significant changes in the gene expression of the inflammatory mediators, interleukin 1β (*IL-1β*), tumor necrosis factor-*α*, *IL-6*, *IL-17A*, interferon-*γ*, or *IL-10*; however, the expression of transforming growth factor β was significantly increased in the first week. In summary, GO-CT sutures may have potential as a suture material in the clinic.

## 1. Introduction

Although there are many methods for the closure of mechanical traumas, such as bandages and adhesives, surgical sutures are still the most widely used for wound closure [1]. The application of surgical sutures can be traced back to 4000 years ago. Suture technology developed rapidly in the past 200 years and has become the largest class of biomedical materials with a market value of $1.3 billion annually [2]. With the development of synthetic macromolecule materials, such as polyglycolide (PGA), in the 1970s, synthetic macromolecule absorbable sutures developed vigorously and achieved great commercial success [3]. The synthetic macromolecule absorbable surgical suture has obvious advantages over conventional non-absorbable suture, which can degrade in vivo after completing its function. However, as a foreign matter, the synthetic macromolecule materials would inevitably cause tissue inflammation to some extent in vivo, which gradually attracts the attention of researchers and clinicians [4]. Therefore, it is still necessary to find an ideal degradable surgical suture material, such as chitin.

Chitin (β-(1-4)-poly-*N*-acetyl-d-glucosamine) is widely distributed and is the second most abundant polysaccharide after cellulose in nature [5]. Previous studies in vivo have indicated that chitin and its derivatives are beneficial for wound healing at all stages during the healing process [6,7]. Chitin possesses native biocompatibility, complete biodegradability, no toxicity, non-immunogenicity, anti-scarring activity, and broad-spectrum antibacterial properties, and is widely used in the medical field [8,9]. For example, chitin can be used as a component of medical biomaterials, such as hemostatic agents, immune promoters, tumor inhibitors, injectable biomaterials, drug-delivery vehicles, and healing agents [10,11]. Academic research studies show chitin has a tremendous potential as a suture material [12]. However, due to the inert molecular structure of chitin, it cannot be dissolved in common solvents [13]. Chitosan is a derivative of the deacetylation of chitin, the acetylation degree of which is lower than 50% [14], that has good solubility, however, chitosan surgical sutures can cause tissue inflammation, and its mechanical strength is too weak [2]. Chang et al. [15] reported that chitin exhibited good mechanical strength, a stable structure, and better compatibility than its derivatives, such as chitosan.

For a long time, only a few solvents, such as LiCl/dimethylacetamide (DMAc) mixtures, N-methylmorpholine oxide (NMMO), paraformaldehyde/dimethylsulfoxide (PF/DMSO), and CaCl_2_-MeOH, had been developed to dissolve chitin to prepare materials [16,17]. However, these solvents are often toxic or corrosive. This was a stumbling block to the potential utilization of chitin [14,18,19]. Recently, novel and efficient solvent systems, such as 1-ethyl-3-methylidazolium acetate [C_2_min][OAc] and alkali/urea (or thiourea), were developed to dissolve chitin [20,21], which may promote the application of chitin materials. Despite numerous advantages and unique properties, the mechanical strength of chitin materials is still relatively poor [22]. In order to enhance the mechanical strength of chitin, it is usually prepared in composition materials with other components. Shamshina et al. reported chitin-poly (lactic acid) fibers, which have higher strength than pure chitin fibers (112 vs. 71 MPa) [23]. In an unpublished work, we prepared pure CT sutures with good tensile strength using pure formic acid as the solvent, and such sutures could withstand the strength of the abdominal muscles of rats. However, it is still necessary to increase the mechanical strength of CT when suturing areas need to bear high tensile strength.

An effective way to enhance the mechanical strength of biopolymers is to apply crosslinking or nanofillers [24]. In recent years, graphene-based materials have interested many scientists worldwide because of their extraordinary mechanical, thermal, and electronic properties [25,26,27]. Among the graphene derivatives, graphene oxide (GO) may be the most important and has shown great promise for biomedical applications, such as drug/gene delivery [28], biological sensing and imaging [29], enzyme immobilization [30], and antibacterial activity [31,32]. GO has also been shown to exhibit excellent biocompatibility [33,34]. Kurapati et al. [35] found that myeloperoxidase derived from human neutrophils in the presence of a low concentration of hydrogen peroxide completely metabolize highly dispersed GO. Liang et al. [36] reported that the injection of high doses of GO (25 mg/kg mouse) via the caudal vein showed very low toxicity for male reproduction. In addition, compared to pristine graphene, GO is rich in hydrophilic functional groups, such as –COOH and –OH, which makes it easily dispersed in some polar solvents and to form intercalated compositions with polar polymers through strong hydrogen-bonding interactions [33,37]. Recently, the incorporation of GO nanofillers has led to a variety of polymers with enhanced mechanical properties, such as GO/chitosan [38], GO/gelatin [39], and GO/DNA [40]. The advantages of GO motivated us to explore the possibility of GO as reinforcement in GO and chitin (GO-CT) absorbable surgical sutures.

This study fabricated a novel monofilament GO-CT surgical suture (diameter 0.30–0.35mm, USP2-0) using chitin and graphene oxide, and epichlorohydrin as a crosslinker. Our unpublished work found that after 16 weeks, GO-CT sutures implanted into rat leg muscles were completely degraded. The breaking strength (183.14 ± 29.87 MPa) of GO-CT sutures is comparable to that of multifilament polyfilament acylated chitin sutures of the same diameter (189.95 ± 15.99 MPa) [4]. The structure of GO-CT was examined and characterized by FT-IR and scanning electron microscopy (SEM). Moreover, biocompatibility was studied in vitro and in vivo to evaluate their potential application in the clinic.

## 2. Results

### 2.1. Preparation of GO-CT Sutures

**Figure 1:**
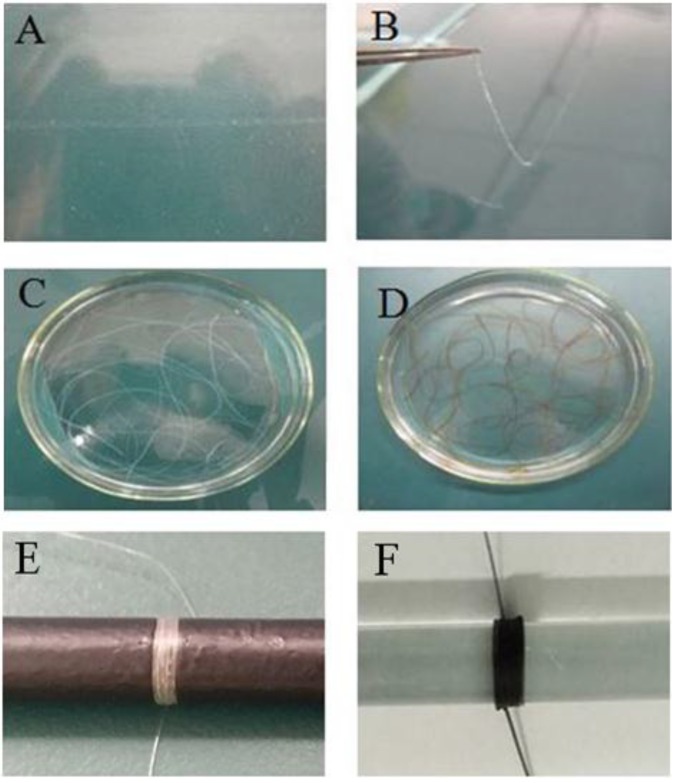
The suture fabrication processes. (**A**) Chitin gel spun into a coagulation bath; (**B**) suture solidification molding; (**C**,**D**) stretched CT and GO-CT sutures in an elution bath, respectively; (**E**) CT suture; (**F**) GO-CT sutures. CT, chitin; GO, graphene oxide.

Figure 1 shows the preparation processes of monofilament surgical sutures of CT and GO-CT. The suture length of CT and GO-CT can be controlled as needed. The length of each suture prepared in this study was 50 cm.

### 2.2. Structure of GO-CT Sutures

**Figure 2:**
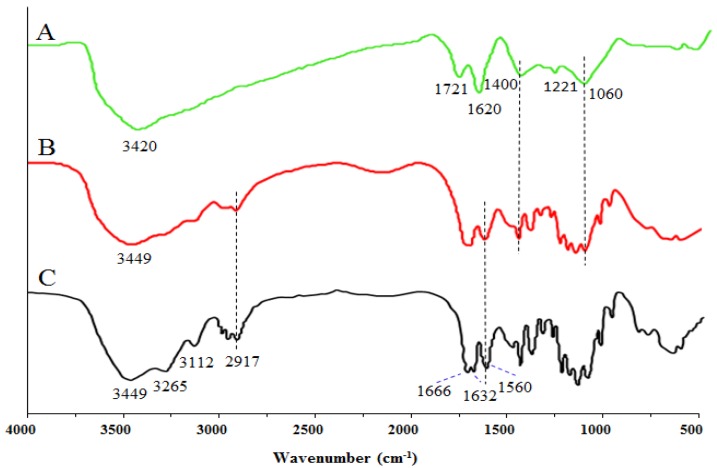
FT-IR spectra of (**A**) GO, (**B**) GO-CT suture, and (**C**) purified CT suture.

Figure 2 shows the spectra of GO, purified CT, and the GO-CT composite suture. In the spectrum of GO, the peak at 3420 cm^−1^ is assigned to the -OH stretching vibration. The strong peak at 1721 cm^−1^ corresponds to the stretching vibration of the C=O carboxylic group. The peak at 1620 cm^−1^ is attributed to the skeletal vibrations of the C=C bond. The peaks at 1400, 1221, and 1060 cm^−1^ are characteristic of other C-O stretching vibrations. In the spectrum of purified chitin, the peak at 3449 cm^−1^ is the -OH stretching vibration, at 3265 cm^−1^ is -NH, and at 1666 and 1632 cm^−1^ is amide band I, and at 1560 cm^−1^ is amide band II. In the spectrum of GO-CT, the peaks at 3449 cm^−1^ are assigned to the -OH groups in GO and CT. The peaks at 1620, 1400, 1221, and 1060 cm^−1^ correspond to the C=C and C-OH/C-O-C groups from GO, respectively.

**Figure 3:**
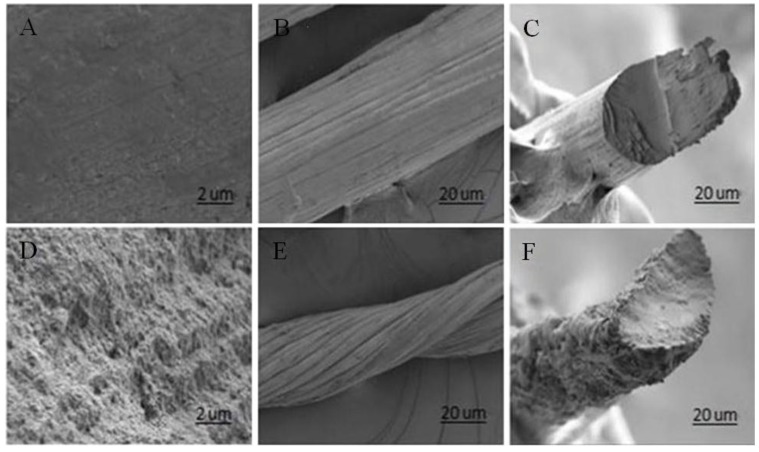
SEM images of GO-CT suture sections with 1.3-fold stretching in (**A**), (**B**), and (**C**) and their corresponding suture section SEM images without stretching in (**D**), (**E**), and (**F**).

Figure 3 shows the SEM images of GO-CT sutures. After being stretched by 1.3-fold, the GO-CT suture has a smoother appearance and the molecular chain orientation of GO-CT becomes more uniform.

### 2.3. Physicochemical Properties of GO-CT Sutures

Table 1 shows the main physicochemical results of the GO-CT sutures according to the absorbable surgical suture national standards of China (YY 1116-2010). The results meet the relative requirements.

Table 2 shows that the breaking strength, knot strength, and knot-pull strength of GO-CT sutures were significantly higher than those of CT sutures (*p* < 0.05), which indicates that the addition of GO can enhance the mechanical strength of CT sutures.

### 2.4. Cytotoxicity Assay

**Figure 4:**
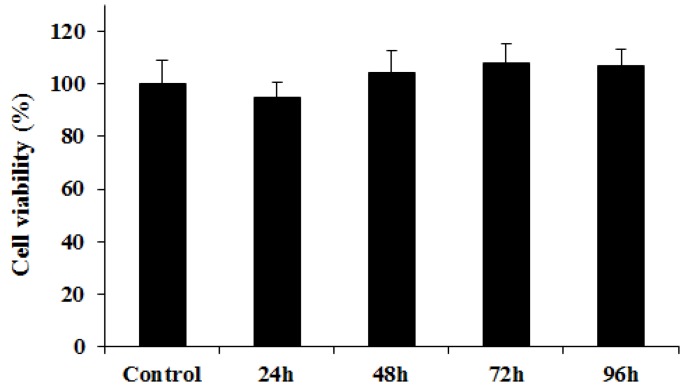
Analysis of GO-CT suture cytotoxicity.

Figure 4 shows the cytotoxicity results for GO-CT sutures obtained by the Tetrazolium salt (MTT) colorimetric method using L929 cells. At 24 h, the relative increment rate of GO-CT cultured cells was 94.62%, and the cytotoxicity grade was grade 1, while at 48, 72, and 96 h, the cytotoxicity grade was grade 0. In general, the cell viability values during the test were all above 90%, indicating that the GO-CT suture had good biocompatibility with L929 cells.

### 2.5. Histological Observation

**Figure 5:**
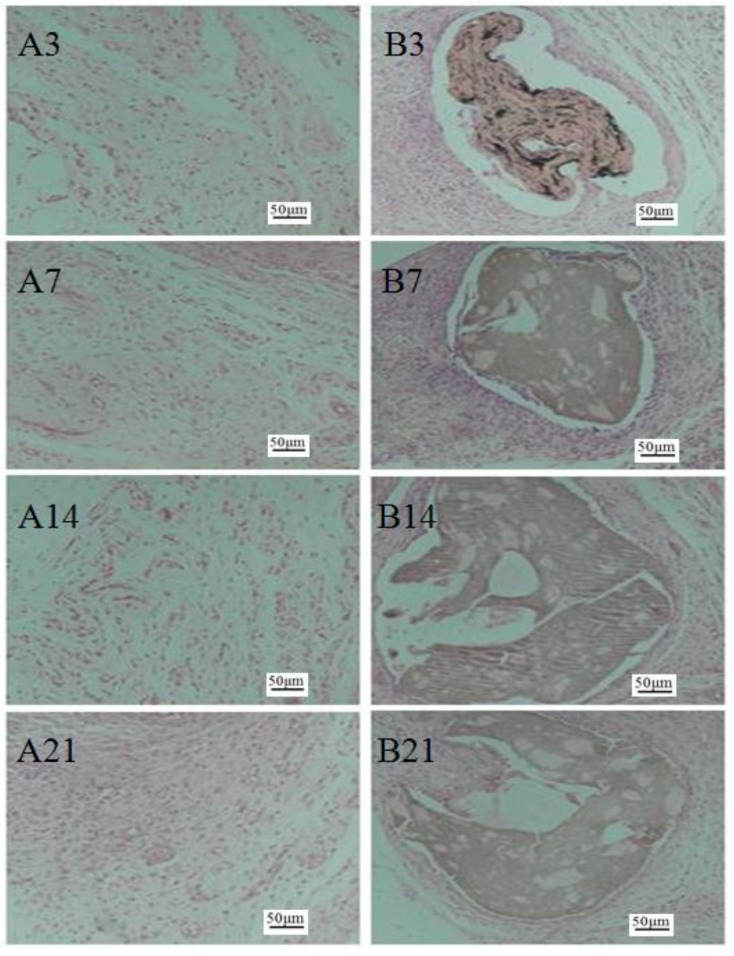
Histological observation of rat dorsal skin transection (hematoxylin-eosin staining). (**A3**–**A21**) Skin section of the control group at 3, 7, 14, and 21 days; (**B3**–**B21**) skin section of the GO-CT group at 3, 7, 14, and 21 days. The ruler size is 50 μm.

As shown in Figure 5, compared to the control group, the GO-CT group did not show an obvious inflammatory response. With the increasing implantation time, subcutaneous tissue gradually fused with the GO-CT suture, which indicates that the GO-CT suture had good biocompatibility in vivo.

### 2.6. Gene Expression of Inflammation-Related Factors

**Figure 6:**
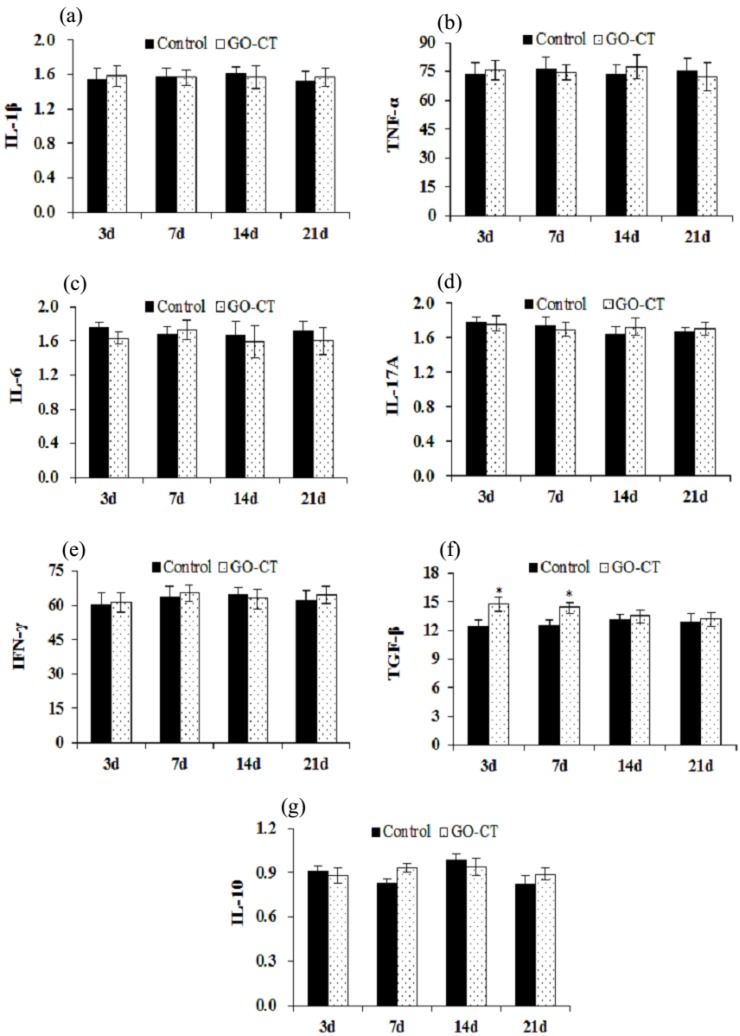
Detections of gene expressions of pro-inflammatory factors tumor necrotic factor-α (TNF-α) (**b**), interleukin 1β (IL-1β) (**a**), iinterleukin 6 (IL-6) (**c**), interleukin 17A (IL-17A) (**d**), and interferon-γ (IFN-γ) (**e**), and anti-inflammatory gene expression transforming growth factor-β (TGF-β) (**f**) and interferon 10 (IL-10) (**g**) at each time point during the experiment. β-Actin is the internal control. d, day.

Figure 6 shows that the pro-inflammatory gene expression levels of IL-1β, TNF-α, IL-6, IL-17A, and IFN-γ showed no significant differences (*p* > 0.05). The anti-inflammatory gene level of TGF-β was significantly increased at 3 and 7 days (*p* < 0.05). However, the gene expression level of IL-10 presented no significant differences (*p* > 0.05).

## 3. Discussion

In this study, a novel monofilament absorbable surgical suture, named GO-CT, was obtained, which had significantly higher mechanical strength than the pure CT suture. Previous studies indicated that chitin could be easily esterified with epichlorohydrin in the presence of NaOH solution [41] and that the hydroxyl groups of chitin and GO can interact with epichlorohydrin through etherification in chitin/GO solution [24]. The detection of the FT-IR spectra of GO-CT sutures suggests the successful combination of GO with CT [42,43].

In addition, the fabrication process may affect the mechanical strength of sutures. Commonly, the aspect ratio of building blocks and their alignment in the fiber axis are the main factors to improve the mechanical performance of polymers [44,45,46]. This study found that by stretching, the orientation of suture fibers was more consistent, and the appearance of the sutures also became smoother. Sutures with smooth appearance have a smaller friction coefficient and can easily penetrate tissues and reduce the damage of sutures to tissues. Moreover, the monofilament fabrication process can reduce the contact area between the suture and tissue fluid and, thus, decrease the risk of infection [47]. However, most monofilament sutures have a limited “memory”, i.e., the ability to restore their original shape. Compared with multifilament sutures, they have poor flexibility and knot strength and are not easy to operate [4]. The GO-CT suture prepared in this study had relatively higher knot strength and scare memory. Overall, the GO-CT suture has the potential to be developed for clinical medical application; however, before clinical application, the characteristics of such sutures need to be determined in vitro and in vivo [48,49].

Medical biomaterials must have good biological safety, and cytotoxicity is an important index in medical biological evaluation systems [50]. For in vitro cytotoxicity experiments, the mouse fibroblast L929 cell line is the most widely used. The International Organization for Standardization has recommended L929 cells as one of the standard cells for cytotoxicity experiments [51]. Previous studies reported that GO may have dose-dependent toxicity to cells and animals and that chronic toxicity could induce apoptosis at high doses [52,53]. However, the present study found that the GO-CT suture had no cytotoxicity to L929 cells, suggesting that the GO-CT suture prepared in this study has good biocompatibility.

An ideal absorbable surgical suture should not cause tissue inflammation or rejection. Skin trauma is common and inevitable in surgery and wounds. Wound healing consists of three stages: Inflammation, proliferation, and maturity. Inflammation lasts 1 to 5 days and the proliferation period lasts about 5 to 14 days. After 14 days, the cells mature, the epidermis regenerates, and the epithelium forms [1,54]. This study investigated skin tissue inflammatory responses of a GO-CT suture implanted subcutaneously in rats. As part of the organism defense mechanism, various types of cell secrete factors that trigger the initial inflammatory response as a regulatory signal for body repair.

It is generally accepted that IL-1β, TNF-α, IL-6, IFN-γ, and IL-17A are pro-inflammatory factors and key mediators in different immune responses and in the acute phase of the inflammatory process [55]. IL-1β and TNF-α activate leukocytes and stimulate the secretion of chemokines [56]. IFN-γ plays an important role in the inflammatory process and is also an important activator of macrophages [57]. IL-17A is one of the key cytokines involved in the mammalian inflammatory response, which stimulates epithelial cells, endothelial fibroblastic cells, and macrophages, resulting in the induction of other inflammatory cytokines, such as IL-1β, IL-6, and TNF-α [58]. This leads to the proliferation and accumulation of neutrophils in the innate immune system and the integration of innate and adaptive immunity in vivo. TGF-β and IL-10 are anti-inflammatory factors and important signals in stopping the immune and inflammatory responses and haves potent effects on wound healing processes. TGF-β also antagonizes some lymphocytic responses [59]. IL-10 is the product of alternatively activated macrophages and inhibits the production of TNF-α [60]. Dysregulated IL-10 production contributes to inflammatory diseases [61]. In this study, we found that there were no significant changes in the gene expression of the cytokines, *IL-1β*, *TNF-α*, *IL-6*, *IFN-γ*, *IL-17A*, and *IL-10*, except that the expression of TGF-β increased significantly in the first week. This indicates that GO-CT sutures have good biocompatibility in vivo and may have a healing-promoting effect.

In summary, as a prepared novel surgical suture, GO-CT exhibited better mechanical strength and biocompatibility in vitro and in vivo and has the potential for clinical application. However, many other factors should be considered before the surgical suture is applied in the clinic, including: The suture degradation cycle; the relationship between the suture degradation cycle and the wound tissue repair cycle; the suture effects in different tissues and organs of animals; and the effects on wounds and infection. Following application in the clinic, further investigation should be conducted, such as follow-up observations after use of sutures and patients’ comfort and satisfaction These should be reported subsequently in a following work.

## 4. Materials and Methods

### 4.1. Materials and Regents

Acetylated *β*-chitin (95%) was purchased from Qingdao Biotemed Biomaterials Co., Ltd. (Qingdao, China). GO was purchased from Nanjing Xianfeng Nanomaterials Technology Co., Ltd. (Nanjing, China). The mouse L929 fibroblast cells were purchased from the Institute of Biochemical Cells, Chinese Academy of Sciences (Shanghai, China). All other reagents in the experiment were commercially analytical reagents.

### 4.2. Preparation of CT and GO-CT Sutures

The preparation of chitin with a low degree of deacetylation was as in [62] with slight modification. Specifically, 3% chitin powder was dispersed in 8% NaOH and 4% urea solution at –80 °C for 48 h. Then, the chitin hydrogel was filtered through 300 silk bolting cloth to remove the impurities. After that, the chitin hydrogel was washed to neutral with deionized water, and vacuum dried. About 20% deacetylation of chitin (DDCT) was obtained as the raw material for preparing sutures.

DDCT (3%) was dissolved in formic acid and stirred at room temperature until completely dissolved, after which the colorless and transparent colloidal DDCT solution was obtained. Then, DDCT gel solution was filtered through 400 silk bolting cloth to ensure uniformity followed by vacuum defoaming. Using anhydrous ethanol as a coagulation bath, monofilament CT sutures of different diameters and lengths could be prepared by controlling the size of the syringe needle. Chitin filamentous glue solution was solidified in a coagulation bath for about 10 s. Then, one end of the hydrogel-like CT suture was gently clamped with tweezers and the whole suture was lifted, and washed in deionized water to neutral. Subsequently, one end of the suture was fixed and the other end was stretched vertically by about 1.3-fold length compared to the original length with an appropriate weight and dried at room temperature for 12 h. Finally, the monofilament CT suture was obtained.

The initial procedure for the preparation of the GO-CT suture was the same as that of the CT suture except that after washing the CT sutures to neutral, the CT sutures were put into NaOH/GO (2 wt%/1.6 wt%) solution and cross-linked by epichlorohydrin (ECH, 1:80 M) overnight with stirring. After that, the GO-CT hydrogel-like sutures were washed with deionized water 3 to 4 times. Then, the GO-CT sutures were stretched and dried to the same extent as the CT sutures. In this experiment, USP2-0 (diameter 0.30–0.35 mm) monofilament GO-CT and CT surgical sutures were prepared. The sutures were sealed and stored after being sterilized using a cobalt-ray until use.

### 4.3. Fourier-Transformed Infrared Spectroscopy and Scanning Electron Microscopy

Fourier-transformed infrared spectroscopy (FT-IR) was used to characterize the structure of GO-CT. FT-IR spectra of the samples were acquired on a NEXUE 470 instrument (Nicolet Co., San Mateo, CA, USA). The KBr method was adopted and data analysis was carried out on a Jwstda-32. In order to observe the effect of tension on the GO-CT suture morphology, scanning electron microscopy (SEM) images were recorded using a Hitachi S4800 (Hitachi, Japan).

### 4.4. Mechanical Properties

We determined the breaking strength, breaking elongation, knot strength, and knot-pull strength of the CT and GO-CT sutures using a Universal Testing Instruments AGS-X (Shimadzu, Japan) at a testing speed of 5.0 mm/s. In addition, after incubation in physiological saline at 25 °C for 24 h, the swelling and pliability, and memory characteristics of CT and GO-CT sutures were evaluated.

### 4.5. Cytotoxicity Assay

A sample of GO-CT sutures preserved after sterilization was used to prepare an extract that contained DMEM medium and 10% newborn bovine serum. The mass-volume ratio of the suture to extract was 1 g/mL. The extraction temperature was 37 ± 2 °C. The extracting time was 72 ± 2 h. According to test method of GB/T 16886.5-2017, L929 mouse fibroblast cells in the logarithmic growth stage were selected for 96-well plate cell culture. The density in the cell plate was 4 × 10^4^ cells/mL and 200 μL/well. The cells were cultured at 37 °C and 5% CO_2_ for 24 h. The culture medium was then replaced by 200 μL/well sterilized extract using the normal culture medium as the control. After culturing for 24, 48, 72, and 96 h, the cells were treated with 20 μL/well 3-(4,5-dimethylthiazol-2-yl)-2,5-diphenyltetrazolium bromide (MTT), and cultured for another 4 h. Then, the culture media containing MTT was removed and 200 μL dimethyl sulfoxide was added to each well. Subsequently, the plates were shaken for 10 min at 37 °C and the absorbance was measured at 490 nm (Thermo Scientific Multiskan Go, San Diego, CA, USA). Cytotoxicity classification criteria were as follows: 0, ≥ 100%; 1, 75–99%; 2, 50–74%; 3, 25–49%; 4, 1–24%; 5, 0%. The cell relative growth rate (RGR) was calculated using the equation: RGR = OD_test_/OD_control_ × 100%, where OD_test_ and OD_control_ were the absorbance values of the test and control groups, respectively.

### 4.6. Histological Observation

Forty-eight adult Wistar rats (200 ± 15 g) were purchased from the Medical Experimental Animal Center of Guangdong Province (Guangzhou, China). The animal experiment was conducted one week later when the rats were adapted to the environment in the animal facility (20 ± 1 °C, humidity 40–60%, 12/12-h light-dark cycle) prior to and during the experimental period. All rats received food and water ad libitum.

When the experiment began, 48 rats were divided equally into the control and GO-CT groups. Four testing time points (3, 7, 14, and 21 days) after the implantation were used in this experiment. Six rats from each group were randomly selected at each testing time. Animals that died during the experiment were necropsied immediately to detect whether there were pathological changes in their viscera. Rats were anesthetized with an intraperitoneal injection of 3% (w/v) pentobarbital sodium (1 mL/kg) [35]. After fixing their abdomen to the operating table, the rat back was shaved to expose the back skin and sterilized with iodophor, and then deiodized with 75% alcohol. Then, sterilized scalpels and forceps were used to longitudinally open the back skin of all rats. The cut was as small as possible to reduce pain for the rats. GO-CT sutures (10 cm) were implanted subcutaneously in the GO-CT group, while nothing was implanted in the control group and the other operations were the same as the GO-CT group. When the operation was finished, benzylpenicillin (5 million units/kg) was intraperitoneally injected to disinfect.

The animal protocol was approved by the ethics review board of Guangdong Ocean University. All of the procedures were performed in accordance with the standards of National Institutes of Health Guide for the Care and Use of Laboratory Animals (NIH Publications No. 8023, revised 1978) and relevant policies in China.

### 4.7. Sample Collection

At each time point, three rats were selected from the experimental and control groups. Before sampling, all rats were anesthetized using 3% (w/v) pentobarbital sodium (1 mL/kg) by intraperitoneal injection. After the back was shaved and disinfected, the full-thickness skin (1 × 1 cm) was sampled in the operative area. Some skin tissues were stored at –80 °C for preservation before use. The other skin tissues were fixed with Bonn’s solution for hematoxylin and eosin (H&E) staining for histological evaluation.

### 4.8. Quantitative Real-Time PCR

To investigate the biocompatibility of CT and GO-CT sutures, the pro-inflammatory gene expression (tumor necrotic factor-α (TNF-α), interleukin 1β (IL-1β), iinterleukin 6 (IL-6), interleukin 17A (IL-17A), interferon-γ (IFN-γ)), and anti-inflammatory gene expression (transforming growth factor-β (TGF-β) and interferon 10 (IL-10)) were determined. Primers were designed using Primer Premier 5 software (Table 3) and synthesized by Gene Denovo Biotechnology Co., Ltd. (Guangzhou, China). Total RNA was isolated from previously frozen tissues using TRIzol Reagent (Invitrogen, San Diego, CA, USA) according to the manufacturer’s instruction. Melting-curve analysis was performed to determine the target specificity. The qRT-PCR data were calculated using the 2^−ΔΔCT^ method [63] with β-actin as the internal control. 

### 4.9. Statistical Analysis

All data collected from the experiment were subjected to Levene’s test of equality of error variances and one-way ANOVA followed by Duncan’s test using SPSS 22.0 (SPSS Inc, Chicago, IL, USA). Statistical significance was considered at *p* < 0.05.

## Figures and Tables

**Table 1:** The basic physicochemical properties of GO-CT sutures (USP2-0) (*n* = 9).

Suture	Water (%)	Dash (%)	Bacterial endotoxin (EU/mg)	Heavy metal (ppm)
GO-CT	0.03	0.04	<0.20	<6

**Table 2:** The comparison between GO-CT and control of CT sutures (*n* = 9).

Index	Suture	
	GO-CT	CT
Breaking strength (MPa)	183.14 ± 29.87 ^*^	89.93 ± 9.55
Breaking elongation (%)	17.34 ± 2.78	20.43 ± 3.21
Knot strength (MPa)	96.92 ± 10.76 ^*^	43.95 ± 8.07
Knot-pull strength (MPa)	124.66 ± 11.03 ^*^	68.35 ± 7.85
Swelling (%)	108.18 ± 9.88	115.65 ± 10.13
USP size	2–0	2–0
Diameter	0.30–0.35 mm	0.30–0.35 mm
Memory	scare	scare
Formation	monofilament	monofilament
Length	50 cm	50 cm

Note: The asterisk “*” represents significant differences (*p* < 0.05).

**Table 3:** Primers used for real-time quantitative PCR analysis.

Gene	Primer	Sequence (5′-3′)	Amplicon Size (bp)
IL-1β	F	ACAGCAGCACATCAACAAGAG	563
	R	CTTTCATCACACAGGACAGG	
TNF-α	F	TACTGAACTTCGGGGTGATCGGTCC	412
	R	CAGCCTTGTCCCTTGAAGAGAACC	
IL-6	F	GAAATGATGGATGCTTCCAAACTGG	548
	R	CACTAGGTTTGCCGAGTAGATCTC	
IL-17A	F	GCCGAGGCCAATAACTTTCT	204
	R	AGAGTCCAGGGTGAAGTGGA	
IFN-γ	F	ATGAGTGCTACACGCCGCGTCTTGG	420
	R	GAGTTCATTGACAGCTTTGTGCTGG	
TGF-β	F	GTAGCTCTTGCCATCGGG	218
	R	GAACGTCCCGTCAACTCG	
IL-10	F	CAATAACTGCACCCACTTCC	352
	R	ATTCTTCACCTGCTCCACTGC	
β-actin	F	GTGGGGCGCCCCAGGCACCA	416
	R	GTCCTTAATGTCACGCACGATTTC	
